# Whither immunity? The search for effective, durable immunity to coronavirus disease 2019 (COVID-19)

**DOI:** 10.1017/ice.2020.442

**Published:** 2020-09-04

**Authors:** David K. Henderson, Sarah D. Haessler, Mary K. Hayden, David J. Weber, Hilary Babcock, Anurag Malani, Sharon B. Wright, A. Rekha Murthy, Judith Guzman-Cottrill, Clare Rock, Trevor Van Schooneveld, Corey Forde, Latania K. Logan

**Affiliations:** 1 Clinical Center, National Institutes of Health, Bethesda, Maryland; 2 UMass Medical School–Baystate Campus, Springfield, Massachusetts; 3 Rush University Medical Center, Chicago, Illinois; 4 University of North Carolina, Chapel Hill, North Carolina; 5 Washington University School of Medicine, St. Louis, Missouri; 6 St. Joseph Mercy Health System, Ann Arbor, Michigan; 7 Beth Israel Deaconess Medical Center, Boston, Massachusetts; 8 Cedars Sinai Health System, Los Angeles, California; 9 Oregon Health and Science University, Portland, Oregon; 10 Johns Hopkins University School of Medicine, Baltimore, Maryland; 11 University of Nebraska Medical Center, Omaha, Nebraska; 12 Queen Elizabeth Hospital, Barbados

One of the most important and challenging questions facing medicine today concerns the extent to which immunity develops and persists following coronavirus disease 2019 (COVID-19), that is, infection with severe acute respiratory coronavirus virus 2 (SARS-CoV-2), or for that matter, following immunization with a candidate SARS-CoV-2 vaccine. During the first 6 months of the pandemic, a great deal of speculation was expressed about whether immunity would follow infection. One prepublication study that has not yet been peer reviewed has suggested that coronavirus protective immunity has a short duration.^1^ At this stage of the pandemic, whether individuals who recover from COVID-19 can get infected again remains uncertain.^2,3^ Nonetheless, an enormous scientific effort is being expended urgently to develop vaccines and monoclonal antibodies to attempt to mitigate the risk for infection on the assumption that protective immunity can and does develop. Now 8 months into the pandemic, what do we know about immunity to SARS-CoV-2? What we have learned thus far suggests important roles for nonspecific, humoral, and cellular immunity.

## Nonspecific immunity

Several manuscripts have suggested a hypothetical role for nonspecific immunity provided through the interferon network and natural killer (NK) cells^4^ in defending against the virus. Several studies have shown that NK cells are depleted or exhausted in severe COVID-19 infection.^5–7^ Vaccines, such as Bacillus Calmette–Guérin (BCG),^8–11^ measles,^10,12^ measles, mumps, rubella (MMR),^13,14^ and oral polio vaccines,^10^ stimulate nonspecific immunity. One preliminary epidemiological study found that, among studied countries in which BCG vaccination is given at birth, COVID-19 contagion rates were lower. These countries also experienced fewer COVID-19 deaths.^8^

## Humoral immunity

The role of B-cell–mediated humoral immunity has been debated.^15^ Some investigators suggest that the humoral response might be ephemeral and incompletely protective, whereas others have found the presence of neutralizing antibodies and robust antibody responses among recovered patients.^16^ Longitudinal studies of antibody protection from seasonal coronavirus infection have shown transient protection, with frequent reinfections occurring 12 months after infection and substantial decreases in antibody levels within 6 months following infection.^1^ In a study of symptomatic and asymptomatic COVID-19 infections in China, asymptomatic infections produced a weaker, more transient immune response, with diminished IgG and neutralizing antibody levels.^17^ Even if humoral immunity is transient, plasma from patients who have recovered that contains high-titer neutralizing antibody might be beneficial. Many anecdotes describing the successful use of convalescent plasma have been reported, but only 1 controlled trial has been reported—it was underpowered and was terminated before statistical significance could be achieved.^18^ Other large, blinded controlled trials are underway.^19^ A recently posted preprint, which has not yet been peer reviewed, from the large, expanded access trial coordinated by the Mayo Clinic, identified reduced mortality associated with both the administration of higher antibody-titer plasma as well as with earlier administration of the plasma for hospitalized COVID-19 patients.^20^ More recently, investigators have demonstrated that antibodies directed against the SARS-COV-2 spike protein are neutralizing and correlate with protection against reinfection in a macaque model.^21^

## Cellular immunity

Several recent papers suggest that cellular immunity likely plays a key role in defense against COVID-19. Lymphopenia occurs commonly, especially in patients who have severe infections, and the severity of the lymphopenia correlates directly with the severity of disease. Lymphopenia in COVID-19 is associated with the depletion of both CD4 and CD8 T cells, with minimal change to the CD4:CD8 ratio.^22^ Whether lymphopenia relates to the sequestration of T lymphocytes in sites of active infection or active destruction of T cells is not yet completely clear. One investigator has suggested that the varied presentations of the disease in patients with COVID-19 could be related to some extent to CD8+ T-cell memory of other coronaviruses.^23^ Sekine et al,^24^ in a paper that has not yet been peer reviewed, evaluated individuals who had asymptomatic or very mild COVID-19 and found vigorous memory T-cell responses in both populations. Interestingly, they also detected SARS-CoV-2–specific T cells in seronegative family members. This paper did not address the extent to which cross immunity to prior seasonal coronavirus infections may have played a role in their findings.

LeBert et al^25^ demonstrated that COVID-19 induces durable T-cell immunity to a SARS-CoV-2 structural protein. Grifoni et al^26^ assessed the virus to identify aspects of viral proteins that they predicted would likely stimulate T cells effectively. They then exposed cells from 10 recovered COVID-19 patients to these viral protein fragments. All 10 patients had helper T cells that responded to the SARS-CoV-2 spike protein. In addition, 7 of the 10 patients responded to stimulation with these protein fragments by producing virus-specific killer T cells.^26^ Similarly, in a preprinted paper, Braun et al.^27^ found helper T cells that could target the spike protein in 15 of 18 patients hospitalized with COVID-19. These latter 2 papers, as well as a paper by Mateus et al,^28^ identified cells reactive with SARS-CoV-2 proteins in healthy individuals who had not been exposed to COVID-19. Also, 2 of these papers suggest that these responses likely represent cross-reactive T-cell recognition between seasonal coronaviruses and SARS-CoV-2.^26,28^

## Animal challenge studies

Two recently published studies, one peer reviewed^21^ and the other posted as a preprint,^29^ have demonstrated that macaques infected with SARS-CoV-2 are resistant to reinfection with the same viral isolate following recovery from their initial infection. In the former study, macaques that had recovered from a laboratory-induced COVID-19 and were rechallenged with the same inoculum demonstrated a 5 log_10_ reduction in median viral loads in bronchoalveolar lavage and nasal mucosa compared with levels detected during their primary infections. In both studies, high levels of neutralizing antibodies were detected following rechallenge. Neither study assessed the cellular immune responses of the macaques in detail.

## Clinical experience to date

Several studies provide some evidence that clinical infection, and even mild infection, can produce a protective immune response. One study evaluated a COVID-19 outbreak that occurred on a fishing vessel.^30^ The outbreak attack rate was 85% (104 of 122 crew members); however, 3 crew members who were known to have neutralizing antibodies before departure remained uninfected. Thus, these 3 individuals who were known before the outbreak to have robust antinucleoprotein antibody responses and neutralizing antibodies were apparently protected from infection. Two recently preprinted studies that have not yet been peer reviewed have also detected robust neutralizing humoral responses among patients who have recovered from COVID-19.^31,32^ Finally, although some cases of possible reinfection with SARS-CoV-2 have been reported,^33^ such cases are rare, and none of these cases has been definitely proved to represent de novo reinfection. A Centers for Disease Control and Prevention (CDC) website states clearly that they are not aware of any confirmed reports of COVID-19 reinfection occurring within 90 days of the primary infection.^34^ The absence of documented reinfections indirectly argues for at least short-term protective immunity in recovered individuals.

In summary, although far from conclusive, the studies of natural, humoral, and cellular immunity, when considered in context with the findings from the animal rechallenge studies, provide substantial encouragement for the concept that recovery from COVID-19 is associated with a robust immune response that includes both humoral and cell-mediated responses. We will not know definitively whether prior infection or vaccine response confers immunity until a sufficient number of previously infected or vaccinated persons are exposed to the virus. The animal rechallenge studies suggest a likely protective host response. In support of this latter concept, thus far into the pandemic, few, if any, cases of COVID-19 reinfection have been documented. Altmann and Boyton^35^ have argued that, based on experience with both SARS-CoV-1 and MERS, humoral responses may be relatively short-lived, and that T-cell responsiveness is potentially more durable. Recently, investigators have found durable T-cell help in those recovered from COVID-19.^26^ Taken together, all these findings point optimistically toward the development of successful vaccines against COVID-19.

## Notes

*Since this manuscript was submitted, a few cases of SARS-CoV-2 re-infection have been well documented.^36–38^ Based on the experience with seasonal coronaviruses, such reinfections were anticipated. The fact that such cases are documented infrequently provides indirect evidence for durable immunity.
